# Fast Microwave-Assisted Hydrothermal Synthesis of Pure Layered δ-MnO_2_ for Multivalent Ion Intercalation

**DOI:** 10.3390/ma11122399

**Published:** 2018-11-28

**Authors:** Martin Eckert, Willi Peters, Jean-Francois Drillet

**Affiliations:** DECHEMA Forschungsinstitut, Theodor-Heuss-Allee 25, 60486 Frankfurt am Main, Germany; martin.eckert@dechema.de (M.E.); peters@dechema.de (W.P.)

**Keywords:** aluminum-ion battery, rechargeable aluminum battery, cathode materials, electrical energy storage, zinc-ion battery, manganese oxide, hydrothermal, microwave-assisted, intercalation electrode material

## Abstract

This work reports on the synthesis of layered manganese oxides (δ-MnO_2_) and their possible application as cathode intercalation materials in Al-ion and Zn-ion batteries. By using a one-pot microwave-assisted synthesis route in 1.6 M KOH (Mn_VII_:Mn_II_ = 0.33), a pure layered δ-MnO_2_ *birnessite* phase without any *hausmannite* traces was obtained after only a 14 h reaction time period at 110 °C. Attempts to enhance crystallinity level of as-prepared *birnessite* through increasing of reaction time up to 96 h in 1.6 M KOH failed and led to decreases in crystallinity and the emergence of an additional *hausmannite* phase. The influence of Mn_II_:OH^−^ ratio (1:2 to 1:10) on phase crystallinity and *hausmannite* phase formation for 96 h reaction time was investigated as well. By increasing alkalinity of the reaction mixture up to 2.5 M KOH, a slight increase in crystallinity of *birnessite* phase was achieved, but *hausmannite* formation couldn’t be inhibited as hoped. The as-prepared layered δ-MnO_2_ powder material was spray-coated on a carbon paper and tested in laboratory cells with Al or Zn as active materials. The Al-ion tests were carried out in EMIMCl/AlCl_3_ while the Zn-Ion experiments were performed in water containing choline acetate (ChAcO) or a ZnSO_4_ solution. Best performance in terms of capacity was yielded in the Zn-ion cell (200 mWh g^−1^ for 20 cycles) compared to about 3 mAh g^−1^ for the Al-ion cell. The poor activity of the latter system was attributed to low dissociation rate of tetrachloroaluminate ions (AlCl_4_^−^) in the EMIMCl/AlCl_3_ mixture into positive Al complexes which are needed for charge compensation of the oxide-based cathode during the discharge step.

## 1. Introduction

The permanent increase in energy storage devices is a very ambitious challenge that obviously cannot be overcome with established lead-acid, NiMH, and Li-ion technologies alone, mostly due to limited supplies of raw material resources and a lack of efficient recycling processes, especially in the case of Li-ion batteries. Therefore alternative electrochemically-active and abundant materials such as Na, Mg, Mn, Al, and Zn are appealing candidates. However, intercalation of multivalent ions into common host matrixes such as manganese oxide spinel is strongly affected by higher diffusion barriers than monovalent ones [1]. Nonetheless, the search for more adapted insertion structures than e.g., Mn_2_O_4_ for zinc and aluminum ion appears to be a very interesting strategy.

The aluminum-ion battery (AIB) is a very promising post lithium-ion battery (LIB) due to the high volumetric energy density of aluminum (~8 Ah L^−1^ vs. 2 Ah L^−1^ for LIB), its well established manufacturing and recycling processes, as well as its global availability and abundance. Thus, the AIB covers all prerequisites to develop a sustainable and ecologically-benign battery. Historically, this concept was limited due to the lack of electrolytes for reversible aluminum deposition and dissolution [2]. In the presence of oxygen and/or water, a thin passivation layer of Al_2_O_3_ is instantaneously formed on the surface of aluminum that can be dissolved by the usage of highly acidic solutions such as AlCl_3_ in EMIMCl or urea. Thirteen years ago, reversible aluminum deposition was successfully carried out by Endres et al. [3,4,5] in EMIMCl/AlCl_3_ ionic liquid (IL). Recently, Abbott [6] & Angell et al. [7] demonstrated aluminum stripping/deposition in urea/AlCl_3_ deep eutectic solvents (DES); kinetics in DES were clearly slower than that in IL electrolyte, so the majority of published investigations on AIB were carried out in an EMIMCl/AlCl_3_ electrolyte. The best result so far was presented by Lin et al. [8] who developed an AIB using an aluminum foil anode, EMIMCl/AlCl_3_ (1:1.3) electrolyte, and a pyrolytic graphite (PG) or graphitic foam cathode. The latter generally implicates anion de-intercalation during discharging step, and more especially in this case, that of large tetrahedral AlCl_4_^−^ (0.53 nm) ions, with an oxidation state of carbon being n < 1, according to

(1) $$C_{x}^{+n}{\left[{\mathrm{AlCl}}_{4}^{-}\right]}_{\left(s\right)}{+e}^{-}\to C_{x}{}_{\left(s\right)}{+\mathrm{AlCl}}_{4}^{-}{}_{\left(l\right)}$$

which further react with Al anode to form aluminum heptachloride according to reaction below:

(2) $${\mathrm{Al}}_{\left(s\right)}{+7\;\mathrm{AlCl}}_{4}^{-}{}_{\left(l\right)}\to {4\;\mathrm{Al}}_{2}{\mathrm{Cl}}_{7}^{-}{}_{\left(l\right)}{+3\;e}^{-}$$

**Figure A1:**
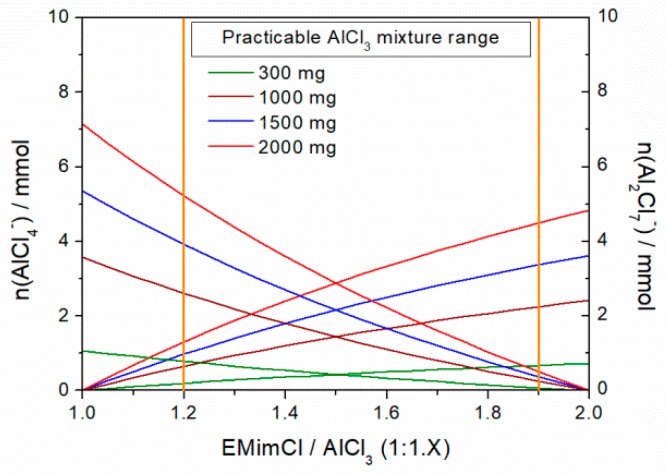
Influence of AlCl_3_ ratio in EMIMCl/AlCl_3_ mixture and electrolyte mass on Al species concentration (calculated values). The practicable molar ratio window lies between 1:1.2 and 1:1.9 (orange lines). At higher AlCl_3_ concentration values, the mixture becomes solid.

Aluminum deposition requires the presence of Al_2_Cl_7_^−^ active species that only exist under acidic conditions. To keep the electrolyte acidic, at least a 1:1.1 EMIMCl/AlCl_3_ molar ratio is required for reversible aluminum deposition/stripping, so that the theoretically-available reversible capacity is defined firstly by an excess concentration of Al_2_Cl_7_^−^ ions in the electrolyte, and secondly, by theoretical maximal insertable anions into the cathode matrix. This AIB battery offers high stability (7000 cycles), even at very high power densities up to 3000 Wh kg^−1^ [8], which is comparable to that of lead acid batteries or super capacitors. However, maximum capacity and energy density is limited to 60–66 mAh g^−1^ and 147–162 mWh g^−1^ normalized to graphite mass [8] due to the large size of solvated AlCl_4_^−^ ion (530 pm) and resulting lower coordination number compared to that of Li^+^ (LiC_6_) in graphite matrix. In this work, we opted for the 1:1.5 EMIMCl/AlCl_3_ mixture that provides equimolar Al_2_Cl_7_^−^ and AlCl_4_^−^ concentration, as can be seen in Figure A1 (Appendix B), and consequently, might deliver higher capacity than the 1:1.3 one. If overall cell energy density is considered, cell performance with the 1:1.5 electrolyte exceeds that of cells with 1:1.3 in all published works.

To circumvent some inconvenient aspects during aluminum stripping/deposition such as passivation, H_2_ evolution, and dendrite formation, the use of an insertion anode material such as TiO_2_ might be an attractive strategy. It should be noted that several published contributions on this material focused mostly on half-cell measurements, and therefore, should be carefully interpreted. Recent investigations reported on reversible Al intercalation into TiO_2_ nanoleaves [9], in which 270 mAh g^−1^ at 50 mA g^−1^ with 8.4% capacity fading over 300 cycles were yielded in a 1 M Al(NO_3_)_3_ aqueous solution. If TiO_2_ is used as the anodic intercalation material, however, cell voltage drop is about 1.10 V vs. an aluminum foil, since Ti^3+^/Ti^4+^ amounts to −0.56 V vs. RHE. Interestingly, Holland et al. recently [10] demonstrated the feasibility of an aqueous Al ion cell under full-cell condition by using 1 M KCl/1 M AlCl_3_ as an electrolyte, anatase TiO_2_ as the insertion anode, and copper hexacyanoferrate (CuHCF) as the cathode. Charge/discharge tests revealed an average discharge capacity of ≈10 mAh g^−1^ with only 7% capacity fade at an average cell voltage of 1.5 V by 333 mA g^−1^ for over 1750 cycles. The authors pointed out that they were not able to elucidate whether the main mechanism charge storage occurs via Al^3+^ intercalation or the surface adsorption reaction. Half-cell measurements of CuHCF as the cathode material and aqueous 0.5 M Al_2_(SO_4_)_3_ as the electrolyte revealed high cyclability but poor capacity (41–22 mAh g^−1^ after 1000 cycles) [11].

In order to increase cell capacity, cobalt-free oxide-based electrodes are preferentially needed. Reversible Al^3+^ intercalation into cathodic VO_2_ nanorods (116 mAh g^−1^, 50 mA g^−1^, 100 cycles) was reported by Wang et al. [12]. A very promising AIB with very high theoretical capacities and energy densities (400 mAh g^−1^, 2.65 V cell voltage, 1060 Wh kg^−1^) was published by Brown et al. [13], who patented a battery system in which good cyclability of cathodic Mn_2_O_4_ spinel in ionic liquid EMIMCl/AlCl_3_ under half–cell conditions is claimed.

Substitution of cobalt in an established LiCoO_2_ intercalation electrode by less expensive and more abundant Mn is an aspired strategy. Meanwhile, manganese oxide-based insertion materials such as layered LiNi_x_Mn_y_Co_z_O_2_ (NMC) are commercially-available cathode materials in LIB for electric vehicles because of their high capacity ≈ 160 Ah kg^−1^, 3.7 V cell voltage, and specific energy ≈ 592 Wh kg^−1^ [14]. Electrochemically active lithium-MOs are mainly divided into three different crystal morphologies: 2D layered (LiMnO_2_, LiCoO_2_ and NMC), 3D spinel (LiMn_2_O_4_), and linear 1D tunnel (LiMnPO_4_). Doped (Cr; Ni; Al; Fe) lithium-MOs are more or less prone to fading, and undergo unwanted growth of solid-electrolyte interface (SEI) or irreversible delithiation, resulting in electrochemically inactive species [15]. However, because of larger and extendable pore sizes compared to spinel and tunnel structures, layered MOs appear to be the most promising candidate for the AIB and ZIB [16].

The feasibility of using MO-based cathodes as insertion materials in zinc-ion battery with a zinc metal anode and an aqueous electrolyte was already demonstrated. Depending on the electrolyte used, (ZnSO_4_ or ZnTfO_2_/H_2_O; ChOAc/ZnOAc/H_2_O), a discharge voltage of 1.6–1.0 V (vs. Zn/Zn^2+^) through both proton and zinc ion intercalation was recorded.

Most of the recently published studies have shown a highly-reversible Zn^2+^ intercalation into tunnel α-MnO_2_ nanorods [17] (233 Ah kg^−1^, 83 mA g^−1^, CE 100%), layered δ-MnO_2_ [18] (252 Ah kg^−1^, 100 cycles) and tunnel α-MnO_2_ (140 Ah kg^−1^, 2000 cycles) [19]. In contrast to water-free δ-MnO_2_ cathodes in non-aqueous organic electrolytes (LIB, etc.), the well-studied *birnessite* crystal structure incorporates water in between the layers. As a consequence, the inter-layer distance increases Å by 0.8, which might be beneficial for zinc ion diffusion.

Synthesis of nanoscale oxides is most favorable at temperatures below 200 °C, which can be achieved by hydrothermal and/or microwave-assisted syntheses. Several different methods have already been published. A good overview of current synthesis routes is given by Wei et al. [20]. δ-MnO_2_ was formed under acidic conditions at 160 °C for at least 24 h [21]. Hydrothermal reaction (24 h, room temperature) of Mn(II) species at different pH (6–11) led to polymorphous crystalline MOs [22]. Amorphous MO nanopowders were synthesized by the reduction of potassium permanganate in organic solvents at room temperature within 2 h [23]. Amorphous nanoscale powders were synthesized by microwave-assisted hydrothermal comproportionation of MnO_4_^−^ and Mn(II) species in neutral aqueous solutions at 75 °C within 30 min [24]. Microwave-assisted hydrothermal reduction of MnO_4_^−^ with NaNO_2_ under acidic conditions led to amorphous MO at 90–170 °C within 8–20 min [25,26] according to following equation

(3) $$\begin{array}{c} {2\;\mathrm{KMnO}}_{4}{}_{\left(\mathrm{aq}\right)}{+3\;\mathrm{NaNO}}_{2}{}_{\left(\mathrm{aq}\right)}{+H}_{2}{\mathrm{SO}}_{4}{}_{\left(\mathrm{aq}\right)}{+2\;H}_{2}O_{\left(\mathrm{aq}\right)}\\ \to {2\;\mathrm{MnO}}_{2}{}_{\left(s\right)}{+3\;\mathrm{NaNO}}_{3}{}_{\left(\mathrm{aq}\right)}{+K}_{2}{\mathrm{SO}}_{4}{}_{\left(\mathrm{aq}\right)}{+3\;H}_{2}O_{\left(\mathrm{aq}\right)} \end{array}$$

Comproportionation reactions of Mn(VII) and Mn(II) species in strongly alkaline media such as NaOH or KOH lead to the formation of sodium *birnessite* Na_0.55_Mn_2_O_4_ * 1.5 H_2_O (PDF 00-043-1456) or potassium *birnessite* K_0.46_Mn_1.54_Mn_0.46_O_4_ * 1.4 H_2_O (PDF 00-080-1098), as studied in detail by Boumaiza et al. [27]. After 16 h reaction time at 60 °C, crystalline K-*birnessite* with some *hausmannite* impurities was obtained. Luo et al. [28,29] found that at a Mn(VII):Mn(II) molar ratio below 0.24, preferentially β-MnOOH (*feitknechtite* PDF 00-018-0804) and Mn_3_O_4_ (*hausmannite* PDF 00-024-0734) are formed, while above 0.40, mostly amorphous material was yielded. They found an optimum at 0.32 molar ratio. Luo also studied the influence of the alkalinity at 0.40 Mn_VII_:Mn_II_ molar ratio on the nature of the MnO_2_ crystal phase, and concluded that at a molar ratio of 0.40 and OH^−^ concentration of ≥1.6 mol L^−1^, the formation of K & Na *birnessite* is favored. Reported reaction times ranged from least 16 h [27] to several days [28,29].

This work aims at both further increasing crystallinity and reducing the reaction time of layered MnO_2_ synthesis by using microwave-assisted hydrothermal comproportionation of MnO_4_^−^ and Mn(II) species in an alkaline solution, according to following reaction

(4) $$\begin{array}{c} 3\;\mathrm{Mn}{\left({\mathrm{NO}}_{3}\right)}_{2}{}_{\left(\mathrm{aq}\right)}{+2\;\mathrm{KMnO}}_{4}{}_{\left(\mathrm{aq}\right)}{+4\;\mathrm{KOH}}_{\left(\mathrm{aq}\right)}\\ \to {5\;\mathrm{MnO}}_{2}{}_{\left(s\right)}{+6\;\mathrm{KNO}}_{3}{}_{\left(\mathrm{aq}\right)}{+2\;H}_{2}O_{\left(\mathrm{aq}\right)} \end{array}$$

## 2. Materials and Methods

### 2.1. Experimental

First, 5.50 g potassium permanganate (Merck, Darmstadt, Germany) was weighed in a glass beaker that was filled with 100 mL ultrapure water (ELGA Purelab©, Veolia Water, Paris, France) to get a stock solution. A 1 g mL^−1^ manganese nitrate (Sigma-Aldrich, Schnelldorf, Germany) solution and a 15.2 mol L^−1^ potassium hydroxide (Merck) solution were prepared in ultrapure water. A typical procedure consisted of pipetting and mixing 10 mL KMnO_4_, 1.8 mL Mn(NO_3_)_2_, 3.9 mL KOH and 21.2 mL ultrapure water.

Microwave-assisted hydrothermal comproportionation was carried out at 110 °C for 14 and 96 h after 1 h aging under vigorous stirring in a microwave (MLS Ethos.lab, Leutkirch, Germany). The reaction mixture was cooled, and then washed with ultrapure water and centrifuged two times. The coating ink was prepared by adding 30 wt% carbon (C65, Emerys, Bironico, Switzerland) and 10 wt% PTFE (Sigma Aldrich, Schnelldorf, Germany), and hereafter air-sprayed on a 36 cm^2^ gas diffusion layer (GDL H2315I2, Freudenberg, Weinheim, Germany). The coated electrodes were punched into disks with a diameter of 18 mm. The electrodes were assembled together with a glass fiber separator (thickness: 1.5 mm), 300 mg of respective AIB or ZIB electrolyte, and an 18 mm metal foil anode disk (Al or Zn). For half cell measurements, a pseudo-reference metal wire (Al or Zn) was applied. All components were assembled into EL-ECC battery cells (El-Cell, Hamburg, Germany). AIB cells were assembled inside a glovebox in an Argon atmosphere (H_2_O and O_2_ < 0.1 ppm)

### 2.2. Characterization Techniques

Thermo gravimetric analysis (TGA) was performed with a NETSCH Jupiter F3 apparatus (Selb, Germany). The samples were heated from 30 to 600 °C with a temperature ramp of 10 °C min^−1^. The nitrogen flow rate was set to 40 mL min^−1^.

XRD analysis was carried out with a BRUKER D8 Advance diffractometer (Billerica, MA, USA) with a goniometer radius of 300 mm and Cu K_α_ radiation (λ = 1.5418740 Å). Standard diffraction patterns were taken at room temperature between 2θ = 10–80° at an increment of 0.02° and 1.5 s exposure time for standard resolution and at an increment of 0.01° and 12 s exposure time for high resolution (HR-XRD) experiments. For XRD analysis at elevated temperatures, a special vacuum oven chamber (HTK1200, BRUKER, Billerica, MA, USA) was used. The temperature profile was increased by 5 °C min^−1^ with a conditioning time of 2 h at three preselected temperatures (250, 400, 600 °C) before high-resolution XRD measurement. XRD patterns were taken between 2θ = 10–80° at an interval of 0.02° with 1.5 s exposure time. All XRD patterns were processed with Match! software (3.5.2.104 version; Crystal Impact, Bonn, Germany).

Scanning electron microscopy (SEM) images were taken with a Hitachi 4100 (Tokyo, Japan) at 20 mm WD and 20 kV to observe morphology of the powder samples.

Specific surface analysis of the powder samples was performed on a Quantachrome Autosorb-iQ-MP-XR (Boynton Beach, FL, USA) with N_2_ as adsorbent.

Since in AIB cells, EMIMCl (1-ethyl-3-methyl-imidazonlinumchloride)/AlCl_3_ is usually used as the electrolyte, the cathode material has to be water-free. Aluminum tetrachloride reacts heavily with residual water under the formation of aluminum hydroxide and hydrochloric acid gas:

(5) $${\mathrm{AlCl}}_{4}^{-}{}_{\left(l\right)}{+3\;H}_{2}O_{\left(\mathrm{aq}\right)}\to \mathrm{Al}{\left(\mathrm{OH}\right)}_{3(s)}{+3\;\mathrm{HCl}}_{\left(g\right)}{+\mathrm{Cl}}^{-}{}_{\left(\mathrm{aq}\right)}$$

To avoid this reaction, and as consequence heavy corrosion induced by HCl gas, all powder samples were dried at 120 °C under a vacuum (as determined in Section 3.5) at the schlenk line (<0.3 mbar) for at least 24 h prior to assembly and tests in laboratory cells.

Electrochemical analysis was carried out in EL-ECC battery cells (El-Cell) with a BioLogic BCS-810 battery testing system (Seyssinet-Pariset, France). Cyclic voltammograms were run at 10 mV s^−1^ and charge/discharge profiles from 2.4 to 0.8 V vs. Al/Al^3+^ in AIB and from 1.8 to 1.0 V vs. Zn/Zn^2+^ in ZIB. Charge/discharge experiments were run at 2.5–30 mA g^−1^ current densities normalized to active cathode mass.

## 3. Results & Discussion

### 3.1. Influence of KOH Concentration on MnO_2_ Powder Synthesis

**Figure 1:**
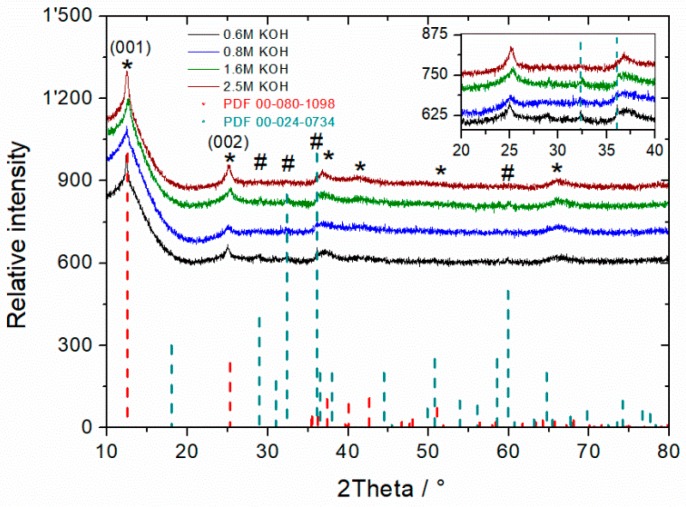
Diffraction pattern of MnO_2_ synthesized at different KOH concentrations and 96 h reaction time. K-*Birnessite* (PDF 00-080-1098 = *) and *hausmannite* (PDF 00-024-0734 = #) are shown as references. Inset: Evaluation of *hausmannite* reflection peaks at 2θ ~32.5° and ~36°.

Formation of qualitative, highly-pure *birnessite* was achieved by Luo et al. [28] after 75 days at room temperature. In order to reduce the reaction time significantly, the influence of temperature and alkalinity on δ-MnO_2_ formation was investigated in this work. In preliminary experiments, the concentration of KOH was varied between 0.6 and 2.5 mol L^−1^, while temperature was fixed to 110 °C and reaction time set to 96 h as shown in Figure 1. The XRD spectra depict as the main phase *birnessite*, the hydrated layered MnO_2._ Due to the low-temperature synthesis which results in low crystalline order and very small particle sizes, the XRD patterns show broad peaks of birnessite. In order to determine the optimal KOH concentration, the main diffraction peak at 2θ = 25° in XRD spectra, which is typical for *birnessite*-type manganese, was first considered and evaluated in terms of intensity and broadening (crystallinity). For the 2.5 M KOH sample, a sharper peak was observed in comparison to those obtained at more diluted concentrations, which indicates more pronounced crystallinity. The higher the molarity, the narrower the (001) peak.

**Figure 2:**
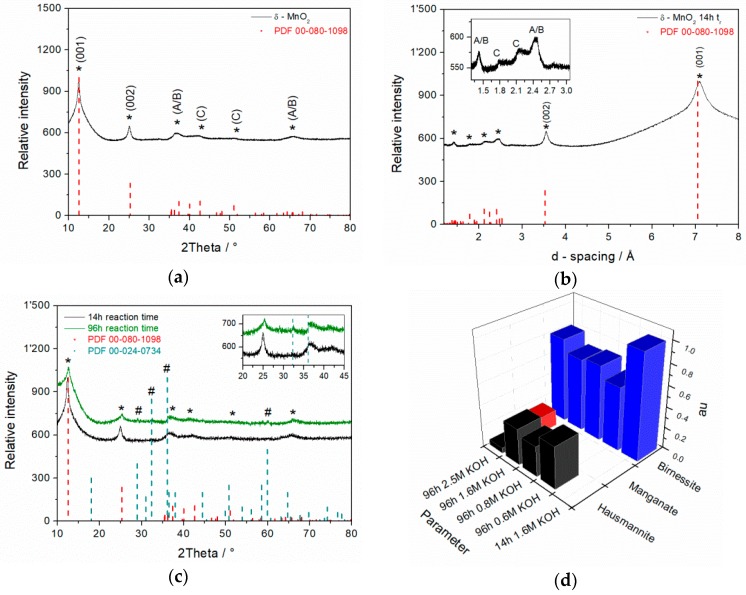
HR-XRD diffraction pattern of as prepared δ-MnO_2_ after 14 h reaction time (**a**,**b**) where, A & B indexes in inlet are related to monoclinic & turbostratic and C to hexagonal crystal system, respectively. Comparative diffraction patterns of MnO_2_ after 96 h and 14 h reaction time (**c**). K-*birnessite* (PDF 00-080-1098 *) and *hausmannite* (PDF 00-024-0734 #) are shown as references. Qualitiative evaluation of MO species in dependence on hydroxide concentration and reaction time from (001) and (002) reflexes (**d**).

Additionally, the formation of *hausmannite*-type manganese phase increased with decrease of alkalinity, so that a compromise between crystallinity and purity level was adopted. Since in 2.5 M KOH, soluble *manganate* species were observed due to incomplete comproportionation reaction, and in 0.6 M KOH highest proportion of *hausmannite* was detected, optimal concentration was set to 1.6 M KOH for further experiments. A qualitative evaluation of the as-synthesized MnO_2_ and 14 h reaction time composition (arbitrary unit = au) based on the peak area of each single phase A_i_ divided by total peak area A_total_ according to following equation (au = A_i_/A_total_) is shown in Figure 2d. Because *hausmannite* has a denser 3D crystal structure (4.59 g cm^−3^) [30] compared to 2D-*birnessite* (3.40 g cm^−3^) with large interplanar space, the *hausmannite* diffraction pattern intensity is significantly more pronounced than that of *birnessite*. Thus, a quantitative determination of phase composition of the as-prepared materials is not straightforward.

### 3.2. XRD Spectra of MnO_2_ Powder Synthesized in 1.6 M KOH for 14 h

HR-XRD measurements of the 14 h/1.6 M KOH sample of as-synthesized MnO_2_ powder revealed characteristics of a highly pure δ-MnO_2_ phase with significant peaks for *birnessite*-type MnO_2_ at 2θ = 12.5° and 25° corresponding to (001) and (002) reflection pairs, respectively, and surprisingly, without any additional phase impurities (Figure 2a,b). The three additional peaks marked with * at higher 2θ values also belonging to birnessite structure cannot be clearly indexed. The d-spacing of the δ-MnO_2_ sheets was determined to 7.1 Å. By plotting d-spacing from HR-XRD measurements, typical reflexes for monoclinic (A) d_200_ ≈ 2.52, d_110_ ≈ 2.48 Å and d_310_ ≈ 1.43 Å, turbostratic (B) d_100_ ≈ 2.42 Å and d_110_ ≈ 1.42 Å and hexagonal (C) d_102_ ≈ 2.0 Å and d_103_ ≈ 1.7 Å crystal systems were identified (Figure 2b) according to the study on the occurrence of different crystal phases in synthetic Na-rich *birnessite* and hexagonal *birnessite* published by Drits et al. [30].

Additionally, the significantly narrower shape of a (001) peak at 2θ = 12.5° is a clear indication for higher crystallinity level after only 14 h reaction time compared to that after 96 h (Figure 2c). This obvious result confirms that the microwave technique is well-suited for the fast synthesis of highly pure *birnessite* manganese oxides.

**Figure 3:**
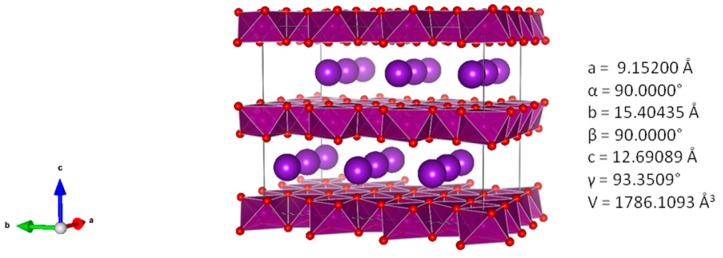
Density functional theory (DFT)-computed polyhedral crystal structure of monoclinic K_0.5_MnO_2_ [31]. Intercalated potassium ions are always localized near to Mn^3+^ positions. The calculated d-spacing value for water-free structure is 6.35 Å. In presence of additional water in-between MnO_2_ layers, typical value amounts to ≈7.1 Å.

The appearance of a predominant monoclinic crystal phase besides the hexagonal one (shown in Figure 2b) might be explained by post-synthesis treatment thereof. Once the residual interlayer water in-between hexagonal layers has been removed during drying step, the potassium ions are anchored in MnO_2_ matrix, as shown in Figure 3**,** and as a consequence, the basal planes of *birnessite* shift along the *b*-axis due to the negative and repellent charge of emergent oxygen atoms from the MnO_2_ plane (Figure 3).

This assumption is in good accordance with the obtained HR-XRD diffraction pattern (Figure 2a,b). In the presence of water, hexagonal crystal phase prevails.

### 3.3. Influence of Hydroxide Concentration on Reaction Kinetics

In order to get a better insight into thermodynamic driving forces of each reaction step, free Gibbs enthalpies were calculated for all considered reactions based on equal concentrations of 1 M at 298 K and given in Table 1.

The concentration of OH^−^ was set to 1.6 mol L^−1^ because at 2.5 mol L^−1^, a stable green colored manganate(VI) solution was obtained as mentioned, according to following reaction steps:

(6) $${\mathrm{Mn}}^{2+}{}_{\left(\mathrm{aq}\right)}{+2\;\mathrm{OH}}^{-}{}_{\left(\mathrm{aq}\right)}\overset{k_{1}}{\to }\;\mathrm{Mn}{\left(\mathrm{OH}\right)}_{2(s)}$$

(6.1) $$\Delta_{r}G=-615\frac{\mathrm{kJ}}{\mathrm{mol}}-\left(-228\frac{\mathrm{kJ}}{\mathrm{mol}}+2\times \left(-157\frac{\mathrm{kJ}}{\mathrm{mol}}\right)\right)=-73\frac{\mathrm{kJ}}{\mathrm{mol}}$$

(7) $$\mathrm{Mn}{\left(\mathrm{OH}\right)}_{2(s)}{+\mathrm{MnO}}_{4}^{-}{}_{\left(\mathrm{aq}\right)}{+\mathrm{OH}}^{-}{}_{\left(\mathrm{aq}\right)}\overset{k_{2}}{\to }{\beta -\mathrm{MnOOH}}_{\left(s\right)}{+\mathrm{MnO}}_{4}^{2-}{}_{\left(\mathrm{aq}\right)}{(\mathrm{green})+H}_{2}O_{\left(\mathrm{aq}\right)}$$

(7.1) $$\begin{array}{c} \Delta_{r}G=\left(-548\frac{\mathrm{kJ}}{\mathrm{mol}}+\left(-501\frac{\mathrm{kJ}}{\mathrm{mol}}\right)+\left(-237\frac{\mathrm{kJ}}{\mathrm{mol}}\right)\right)-\\ \left(-615\frac{\mathrm{kJ}}{\mathrm{mol}}+\left(-447\frac{\mathrm{kJ}}{\mathrm{mol}}\right)+\left(-157\frac{\mathrm{kJ}}{\mathrm{mol}}\right)\right)=-67\frac{\mathrm{kJ}}{\mathrm{mol}} \end{array}$$

Reactions (6) and (7) are favored due to negative free enthalpies, and therefore, an essential indicator for assumed first reaction steps during MnO_2_ formation. Furthermore, Mn(OH)_2_ forms with an excess of OH^−^, a very stable [Mn(OH)_3_]^−^ complex with a stability constant value of 3.98 × 10^16^. Hence, once the solution reaches the OH^−^ saturation level, the complex is in equilibrium with its precipitate Mn(OH)_2_.

(8) $$\mathrm{Mn}{\left(\mathrm{OH}\right)}_{2(s)}{+\mathrm{OH}}^{-}{}_{\left(\mathrm{aq}\right)}\overset{k_{3}}{↔}{\left[\mathrm{Mn}{\left(\mathrm{OH}\right)}_{3}\right]}^{-}{}_{\left(\mathrm{aq}\right)}$$

Reformed Mn(OH)_2_ can further react with MnO_4_^−^ under formation of MnO_4_^2−^ as described before in Reaction (7). *Manganate*(VI) MnO_4_^2−^ subsequently reacts with Mn(OH)_2_ under the formation of β-MnOOH and blue-colored hypomangante(V) MnO_4_^3−^ as follows

(9) $$\mathrm{Mn}{\left(\mathrm{OH}\right)}_{2(s)}{+\mathrm{MnO}}_{4}^{2-}{}_{\left(\mathrm{aq}\right)}{+\mathrm{OH}}^{-}{}_{\left(\mathrm{aq}\right)}\overset{k_{4}}{↔}{\beta -\mathrm{MnOOH}}_{\left(s\right)}{+\mathrm{MnO}}_{4}^{3-}{}_{\left(\mathrm{aq}\right)}{(\mathrm{blue})+H}_{2}O_{\left(\mathrm{aq}\right)}$$

(9.1) $$\begin{array}{c} \Delta_{r}G=\left(-548\;\frac{\mathrm{kJ}}{\mathrm{mol}}+\left(-722\;\frac{\mathrm{kJ}}{\mathrm{mol}}\right)+\left(-237\;\frac{\mathrm{kJ}}{\mathrm{mol}}\right)\right)-\\ \left(-615\;\frac{\mathrm{kJ}}{\mathrm{mol}}+\left(-501\;\frac{\mathrm{kJ}}{\mathrm{mol}}\right)+\left(-157\;\frac{\mathrm{kJ}}{\mathrm{mol}}\right)\right)=-234\;\frac{\mathrm{kJ}}{\mathrm{mol}} \end{array}$$

Hypomanganate has a high oxidation potential of E^0^ = 0.96 V (vs. NHE) and reacts with β-MnOOH (E^0^ = 0.95 V) to *birnessite* type δ-MnO_2_ according to Equation (10):

(10) $${\beta -\mathrm{MnOOH}}_{\left(s\right)}{+\mathrm{MnO}}_{4}^{3-}{}_{\left(\mathrm{aq}\right)}{+H}_{2}O_{\left(\mathrm{aq}\right)}\overset{k_{5}}{↔}{2\;\mathrm{MnO}}_{2(s)}{+3\;\mathrm{OH}}^{-}{}_{\left(\mathrm{aq}\right)}$$

(10.1) $$\begin{array}{c} \Delta_{r}G=\left(2\times \left(-465\;\frac{\mathrm{kJ}}{\mathrm{mol}}\right)+3\times \left(-157\;\frac{\mathrm{kJ}}{\mathrm{mol}}\right)\right)-\\ \left(-548\;\frac{\mathrm{kJ}}{\mathrm{mol}}+\left(-722\;\frac{\mathrm{kJ}}{\mathrm{mol}}\right)+\left(-237\;\frac{\mathrm{kJ}}{\mathrm{mol}}\right)\right)=+106\;\frac{\mathrm{kJ}}{\mathrm{mol}} \end{array}$$

Since standard potentials of MnO_4_^−^ (E^0^ = 0.558 V) and MnO_4_^2−^ (E^0^ = 0.60 V) in alkaline media are not high enough for MnOOH oxidation, the limiting reaction step is the formation of hypomanganate(V), despite relative high exothermic reaction enthalpy value (−234 kJ mol^−1^). In our experiments at OH^−^ = 0.6 and 0.8 mol L^−1^, other manganese oxides like *hausmannite* (Mn_3_O_4_) were yielded (Figure 3). This may result from too low hypomanganate(V) concentrations. We assume that the reaction constant k_4_ of Equation (9) increases with higher [OH^−^] concentration. This assumption is supported by the increase in crystallinity of formed *birnessite* at 1.6, and especially, 2.5 mol L^−1^ (Figure 3).

The law of mass action for Equations (9) and (10) and the calculated endothermic reaction enthalpy (+106 kJ mol^−1^) of MnO_2_ formation explain the extremely long reaction time of several days at room temperature reported by Luo et al., and at least 14 h at 110 °C in microwave synthesis in this work.

(11) $$K_{4}=\frac{\left[\beta -\mathrm{MnOOH}\right]\times \left[{\mathrm{MnO}}_{4}^{3-}\right]{\times [H}_{2}O]}{\left[\mathrm{Mn}{\left(\mathrm{OH}\right)}_{2}\right]\times \left[{\mathrm{MnO}}_{4}^{2-}\right]{\times [\mathrm{OH}}^{-}]}$$

(12) $$K_{5}=\frac{{\left[{\mathrm{MnO}}_{2}\right]}^{2}\times {\left[{\mathrm{OH}}^{-}\right]}^{3}}{\left[\beta -\mathrm{MnOOH}\right]\times \left[{\mathrm{MnO}}_{4}^{3-}\right]{\times [H}_{2}O]}$$

As assumed, the formation of hypomanganate is directly correlated to the concentration of [OH^−^] and [MnO_4_^2−^] (Equation (11)). Due to low hypomanganate, and therefore, low MnO_2_ concentration at 0.6 & 0.8 mol L^−1^ hydroxide, the equilibrium is massively shifted to the educt site (Equation (12)). This might explain the formation of crystalline *birnessite* phase MnO_2_ at [OH^−^] concentrations ≥ 1.6 mol L^−1^. The formation of *hausmannite* at [OH^−^] concentrations ≤ 1.6 mol L^−1^ can be explained by the thermodynamically-favored reaction of residual Mn(OH)_2_ with MnOOH (−46 kJ mol^−1^) according to Reaction (13). The presence of residual Mn(OH)_2_ can be explained by significantly-decreased hypomanganate formation, according to Equation (11). After 96 h of heating, the thermodynamically more stable *hausmannite* structure is formed due to the reduction and collapse of δ-MnO_2_.

(13) $$\mathrm{Mn}{\left(\mathrm{OH}\right)}_{2(s)}{+2\;\mathrm{MnOOH}}_{\left(s\right)}\overset{k_{6}}{\to }{\;\mathrm{Mn}}_{3}O_{4(s)}{+2\;H}_{2}O_{\left(\mathrm{aq}\right)}$$

(13.1) $$\begin{array}{c} \Delta_{r}G=\left(-1283\;\frac{\mathrm{kJ}}{\mathrm{mol}}+2\times \left(-237\;\frac{\mathrm{kJ}}{\mathrm{mol}}\right)\right)-\left(-615\;\frac{\mathrm{kJ}}{\mathrm{mol}}+2\times \left(-548\;\frac{\mathrm{kJ}}{\mathrm{mol}}\right)\right)=\\ \;-46\;\frac{\mathrm{kJ}}{\mathrm{mol}} \end{array}$$

The limiting reaction step is the *hypomanganate* MnO_4_^3−^ formation that is directly dependent on the MnO_4_^2−^ and [OH^−^] concentration. Our experiments confirm that strongly-alkaline environments between 2.5 and 0.8 with an optimum at 1.6 mol L^−1^ and elevated temperatures are required for the fast formation of layered δ-MnO_2_. However, higher temperatures than 110 °C lead to the formation of hausmannite due the collapse of the *birnessite* crystal structure.

### 3.4. TGA Measurements

**Figure 4:**
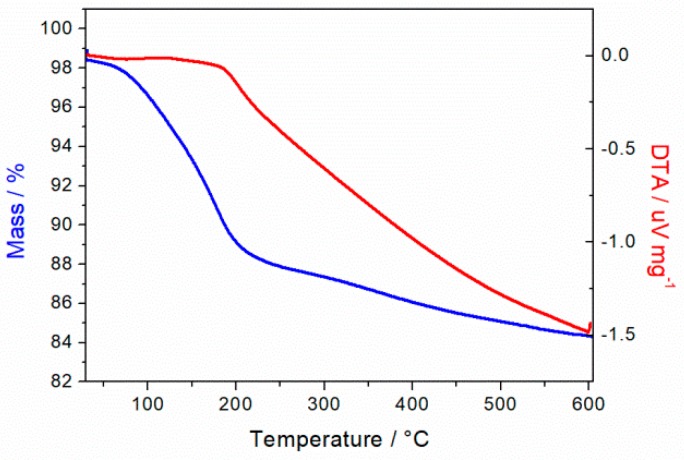
TGA measurements of MnO_2_ powder after synthesis in N_2_ at 40 mL min^−1^, 10 °C min^−^1 heat ramp under atmospheric pressure.

**Figure A2:**
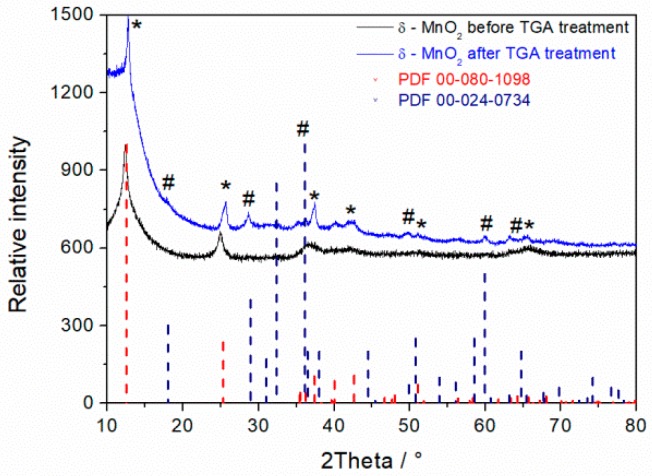
XRD diffraction pattern of MnO_2_ before and after TGA treatment. K-birnessite (PDF 00-080-1098 = *) and hausmannite (PDF 00-024-0734 = #) are shown as references.

TGA measurements were carried out in order to determine the water content of the as-prepared powder material. The initial mass loss of about 10% in a temperature range between 60 and 200 °C is accompanied by a low endothermic change than can be associated with interlayer water evaporation from manganese matrix (Figure 4). At temperatures above 200 °C, a pronounced endothermic process with less intense mass loss is assigned to a crystal phase change from monoclinic to indefinable layered MnO_2_ and *hausmannite* (see XRD spectra in Figure A2 in Appendix B).

### 3.5. In-Situ XRD Spectra

In-situ XRD measurements were carried out to evaluate thermal material stability, and to determine optimal drying parameters for removing all interlayer water, before it can be assembled to an aluminum-ion battery together with the highly water-sensitive electrolyte EMIMCl/AlCl_3_. In-situ XRD measurements took place under a vacuum and small heating steps at a low heating rate (5 °C min^−1^), combined with 2 h equilibration time at respective temperatures of up to 600 °C.

**Figure 5:**
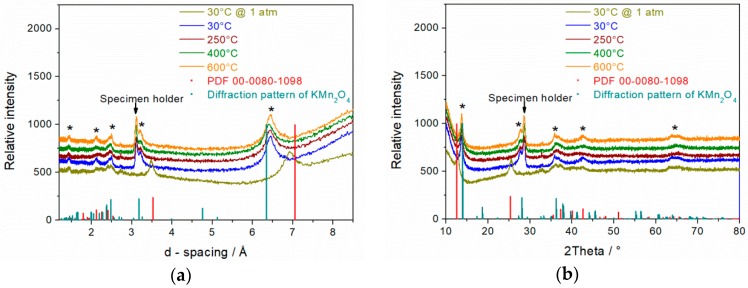
In-situ XRD diffraction pattern of δ-MnO_2_ (14 h @ 110 °C in 1.6 M KOH); All patterns at respective temperatures were recorded under vacuum except 30 °C at 1 atm (**a**,**b**).

The application of a vacuum at 30 °C led to a shrinkage of the d-spacing from 7.1 to 6.45 Å (Figure 5a) that can be correlated to the removal of interlayer water. Further heating to 250, 400, and 600 °C influenced neither the interlayer distance nor the crystal phase. In Figure 3, the d-spacing for the monoclinic structure without any interlayer water amounts theoretically to 6.35 Å. The slightly higher measured d-spacing value of 6.45 Å within 30–600 °C temperature range might be explained by the formation of a *birnessite* polymorph K_x_MnO_2_ with x ≤ 0.5. Thus, the as synthesized δ-MnO_2_ does not fit perfectly with the reference spectra of hydrated or dried single phase monoclinic δ-MnO_2_ (Figure 5b).

Considering that nearly all water can be removed just by applying a vacuum to the sample, we set the drying temperature to 120 °C, slightly above the boiling point of water, to be sure that the whole sample was completely water-free. If a faster drying step is needed, conditioning under a vacuum at higher temperatures would be also feasible.

### 3.6. BET Analysis

**Figure 6:**
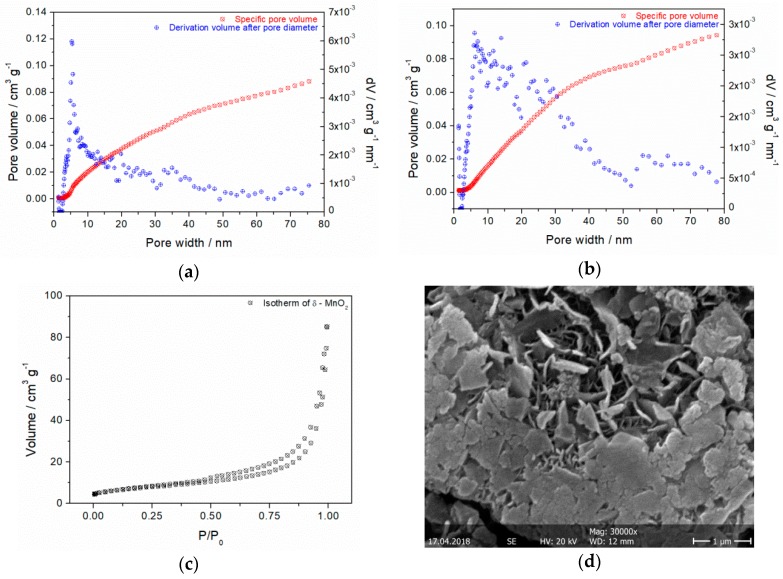
Pore size distribution of δ-MnO_2_ (14 h @ 110 °C in 1.6 M KOH) computed with NLDFT cylindrical pore equilibrium model on silica (**a**), cylindrical pore adsorption branch model (**b**), N_2_ desorption/adsorption isotherms at 77 K for synthesized δ-MnO_2_ after 28.2 h degassing at 150 °C, measuring time 11:41 h:min (**c**) and SEM image of δ-MnO_2_ (**d**).

Specific surface area of as-prepared δ-MnO_2_ powder analysis by N_2_ adsorption revealed a total pore volume of 0.088 cm^3^ g^−1^ and a surface area of 27 m^2^ g^−1^. Hence, the pore size distributions computed for an equilibrium and adsorption model show nearly the same local maxima of mesopores between 5 and 40 nm. The mean pore size was determined to 5.4–6.0 nm (Figure 6a,b). The mesoporous character is confirmed by the presence of hysteresis loop of pore volume for P/P_0_ = 0.5–0.9 in adsorption/desorption isotherm profile shown in Figure 6c. The porous flake-like morphology of δ-MnO_2_ can be seen in Figure 6d. The flakes have a diameter of up to 0.2–2 µm and a thickness of 50–100 nm. These results are in very good accordance with information reported in other works about birnessite type structures [35,36,37,38,39].

### 3.7. Electrochemical Measurements

#### 3.7.1. Aluminum-Ion Battery

**Figure 7:**
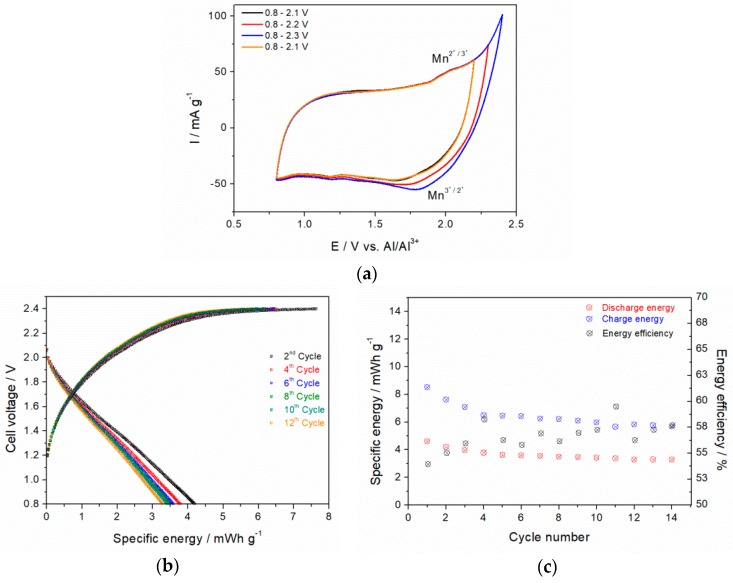
CVs of δ-MnO_2_ (14 h @ 110 °C in 1.6 M KOH) with 18 wt% carbon in 1:1.5 EMIMCl/AlCl_3_ at 10 mV s^−1^ as a function of different potential windows at RT (**a**), galvanostatic charge/discharge behavior of Al-ion cell with an Al foil anode, EMIMCl/AlCl_3_ (1:1.5) as electrolyte and δ-MnO_2_ cathode at 2.5 mA g^−1^ normalized to MnO_2_ mass (**b**) and corresponding specific energy and energy efficiency (**c**).

The influence of a potential window on cyclic voltammograms (CV) profiles of synthesized δ-MnO_2_ in EMIMCl/AlCl_3_ is presented in Figure 7a. The potential window limits were set to 2.45 and 0.80 V vs. Al/Al^3+^ that correspond to upper electrolyte stability window and irreversible manganese reduction at lower potential values than 0.8 V, respectively. A small anodic shoulder at 1.9–2.0 V, and corresponding cathodic peaks shifted from 1.6–1.8 V with increasing the potential window, are induced by the Mn^2+^/Mn^3+^ redox couple. An irreversible reduction peak is observed at 1.2 V. This might result from reduction of the electrode or from electrolyte impurities. Since the first and last scan between 0.8 and 2.1 V are identical, potential extension up to 2.3 V obviously didn’t affect the manganese oxide structure.

Charge/discharge experiments under full-cell conditions revealed poor energy density of 4.2–3.3 mWh g^−1^ which corresponds to ~0.8% of theoretically value (524 mWh g^−1^) based on Mn^+3/+4^O_2_ redox pair (Figure 7b). Within the first 14 cycles, the energy efficiency was about 56%, as shown in Figure 7c.

As determined in Section 3.5, the interlayer distance in dried layered MnO_2_ is ~6.45 Å, which corresponds to a gallery height of ~3 Å, which is the distance in between the layers. However, for successful intercalation, the large AlCl_4_^−^ (ø = 5.5 Å) has to dissociate into Al^3+^ (ø = 1.08 Å) to enter the gallery. We conclude that the central Al^3+^ ion is too strongly shielded by four Cl^−^ anions in the tetrahedral configuration. Consequently, the affinity of AlCl_4_^−^ anions for dissociation into a positive charge species, which is needed for charge compensation of the cathode material during reduction step, is very poor.

In contrast to our oxide cathode, W. Wang et al. reported successful reversible Al^3+^ ion intercalation into a vanadium oxide (VO_2_) tunnel structure that showed significantly higher capacities in EMIMCl/AlCl_3_ (116 mAh g^−1^ @ 0.9–0.1 V vs. Al/Al^3+^ for 100 cycles) [12]. However, the reported discharge voltage is significantly lower than in this work (2.0–0.8 V). In a similar work, H. Wang et al. claimed that the excellent reversible capacity of 239 mA g^−1^ with a V_2_O_5_ cathode can be attributed to the presence of fully dissociated Al^3+^ ions in the electrolyte [40]. In both works, no proof for the presence of positive Al^3+^ ions in the electrolyte and/or oxide matrix was provided by e.g., Raman, NMR or XPS analysis, respectively.

#### 3.7.2. Zinc-Ion Battery

In contrast to the AlCl_4_^−^ ions in the AIB electrolyte, fully dissociated Zn^2+^ ions (ø = 1.48 Å) are present in aqueous electrolytes. Additionally, as shown in Section 3.5, hydrated layered MnO_2_ has an increased interlayer distance of 7.1 Å, resulting in an accessible gallery height of ~3.8 Å. Thus, the aqueous ZIB appeared to be a priori a more suitable system to demonstrate the MnO_2_ ability for multivalent ion intercalation.

**Figure 8:**
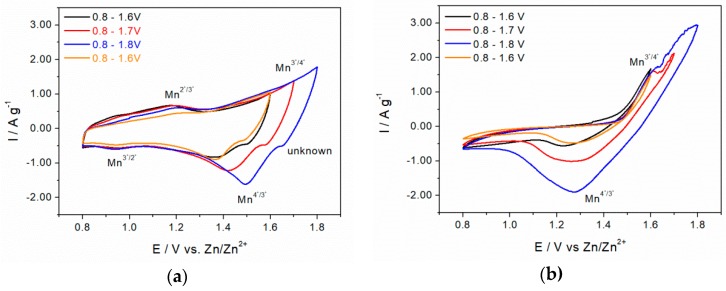
Influence of potential window on δ-MnO_2_ (14 h @ 110 °C in 1.6 M KOH) voltammogram at 10 mV s^−1^ in ChOAc with 0.01 M ZnOAc and 30 wt % H_2_O at 10 mV s^−1^ (**a**) and in 1 M ZnSO_4_ (**b).**

Influence of a potential window on the electrochemical behavior of δ-MnO_2_ in choline/zinc acetate and zinc sulfate electrolytes is shown in Figure 8a,b, respectively. In choline acetate, first cathodic peaks were detected at 1.2 V with the corresponding anodic reduction peak at 0.95 V and attributed to Mn^2+^/Mn^3+^. Above 1.6 V, the current starts to increase rapidly, indicating the deintercalation of H^+^ and Zn^2+^, as well as possible water splitting. The corresponding cathodic peaks shifted between 1.37–1.5 V. An unidentified small reduction peak was observed at 1.6–1.7 V. The overall specific current is significantly higher compared to that of the AIB (0.8–1.8 A g^−1^ vs. 50–110 mA g^−1^). Interestingly, in contrast to AIB-CVs in Figure 7a in which 1st and 4th curves are almost identical, change in CV shape between 1st and 4th curve after successively extending and narrowing the potential window down to 1.5 V in zinc-containing electrolyte is obvious, especially at 1.2 V. This may be an indicator for irreversible structural change in MnO_2_ matrix.

The redox couple of Mn^3+/2+^ visible in choline acetate electrolyte (Figure 8a) was not detectable in CV with zinc sulfate electrolyte (Figure 8b) also down to 0.2 V (not shown here). Since Zn^2+^ concentration in choline acetate is lower by a factor of 100 compared to that in zinc sulfate solution, this redox pair was not observed obviously due to a large overpotential arising from the stronger solvation of Zn^2+^ with SO_4_^2−^ compared to single-charged acetate anions.

At 1.65 V and 1.25–1.3 V, the Mn^3+/4+^ redox pair was observed. The measured reduction potential at ≈1.3 V is in very good accordance with published de-intercalation potential (≈1.3–1.4 V) values of Zn^2+^ from MnO_2_ matrix [41]. The measured currents are even higher than those in ChoAc, and reached almost 2 A g^−1^ at 1.6 V, which may be related to a higher zinc concentration in sulfate solution. The blurred potential above 1.68 V might be explained by oxygen evolution due to water decomposition. The change in CV shape between the 1st and 4th curves indicates a structural change in MnO_2_ matrix (Figure 8b) as observed in ChoAc electrolyte (Figure 8a).

In order to allow easier comparison between AIB and ZIB cyclic voltammograms, redox peak potential regions were normalized to the standard hydrogen reference electrode (SHE), and listed in Table 2. We assume that anodic peaks between 1.96 and 2.03 V and cathodic peaks at 1.66–1.82 V from the AIB experiments correspond to Mn^3+^/Mn^2+^ and not to the oxidation/reduction of Mn^4+^/Mn^3+^ in δ-MnO_2_. The theoretical Mn^4+^/Mn^3+^ redox potential in AIB derived from the measurements in ZIB should be in the range of 2.3–2.7 V (see Table 2), whereas the decomposition onset potential of EMIMCl/AlCl_3_ is ≈2.5 V (vs. Al/Al^3+^). Because of electrolyte decomposition and poor dissociation of AlCl_4_^−^ into positively-charged species, EMIMCl/AlCl_3_ appeared to be an inappropriate electrolyte for high-energy AIB batteries with metal oxide cathodes.

**Figure 9:**
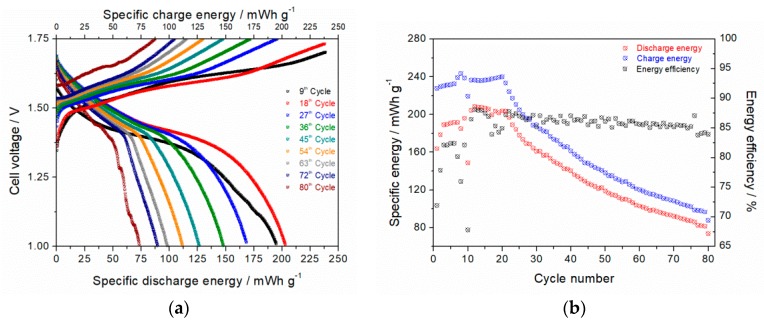
Influence of cycling on charge/discharge behavior of Zn-ion cell at 30 mA g^−1^ in 1 M ZnSO_4_ with δ-MnO_2_ (14 h @ 110 °C in 1.6 M KOH) as cathode and Zn powder as anode.

Results from galvanostatic charge & discharge experiments of zinc-ion cell with δ-MnO_2_ as the cathode material are shown in Figure 9a. After the initiation phase within the first twenty cycles, the specific discharge energy was about 200 mWh g^−1^ with an energy efficiency ≥ 83%. This specific energy corresponds to about 38% of the theoretical value of pure MnO_2_ equal to ~527 mWh g^−1^ by assuming a one-electron transfer step based on the Mn^4+/3+^ redox reaction. The mean discharge voltage was around 1.48 V. The discharge curve shows a plateau region till 1.38 V. As published by Sun et al. [40], this flat plateau region is induced by H^+^ insertion (120–140 mWh g^−1^) from the slightly acidic electrolyte (pH~4.7). Afterwards, the discharge curve became significantly steeper due to insertion of bigger Zn^2+^ ions into MnO_2_ matrix (70–80 mWh g^−1^) at ≤1.35 V. This voltage value is in very good accordance with measured ≈1.3 V intercalation potential as shown in Figure 8b. The potential drop could be explained by a higher energetic diffusion barrier of solvated zinc ions within layered MnO_2_ matrix compared to that of protons. Since the ionic radii of K^+^ (138 pm) and H_2_O (2.78 Å) defines the interlayer distance in δ-MnO_2_ matrix, hydrogen (53 pm) and zinc (74 pm) can be a-priori intercalated. The gravimetric energy and power density was determined to 210 Wh kg^−1^ (748 Wh L^−1^) and 44.4 W kg^−1^. After the 20th cycle, the capacity started to decrease continuously down to 72 mWh g^−1^ after the 80th cycle (Figure 9b). After the 50th cycle, the energy density amounted to 110 Wh kg^−1^ (392 Wh L^−1^) which is ~48% less than that yielded during the first cycle.

## 4. Conclusions

A modified microwave-assisted hydrothermal route based on comproportionation of potassium permanganate and manganese nitrate at 110 °C within 96 h led to layered δ-MnO_2_ *birnessite* phase with *hausmannite* Mn_3_O_4_ as side phase. The optimum KOH concentration in terms of *hausmannite* impurities was found to be 1.6 mol L^−1^. Further efforts to reduce side-phase were very successful by only reducing reaction time down to 14 h. Experiments with shorter reaction time failed. These should be eventually performed at slightly higher temperature than 110 °C.

The as-synthesized manganese oxide powders have been tested as cathode material for both Al-ion and Zn-ion battery. In AIB, a very poor capacity was yielded due to large size (ø = 5.5 Å) and negative charge of AlCl_4_^−^ in EMIMCl/AlCl_3_ electrolytes which does not fit into the gallery of dried MnO_2_ (d = 3 Å). It must be concluded that dissociation of the AlCl_4_^−^ into the much smaller Al^3+^ ions (d = 1.08 Å) didn’t take place due to the strong shielding by chloride ions. In ZIB, the fully-dissociated Zn^2+^ ions (d = 1.48 Å) are readily able to intercalate into the hydrated interlayer space (d = 3.8 Å). A specific discharge energy of about 200 mWh g^−1^MnO_2_ at 1.48 V mean voltage and 85% energy efficiency was obtained within first 20 cycles in 1 M ZnSO_4_. At C5 discharge current, capacity is induced by first proton intercalation between 1.65 and 1.3 V, followed by Zn intercalation at lower cell polarization values. Thus, the as-synthesized δ-MnO_2_ is active for divalent-ion intercalation, and is a very promising candidate as cathode material in Zn-ion batteries. However, cyclability and cathode material conductivity and stability have to be significantly improved.

## Figures and Tables

**Table 1:** Overview of standard free Gibbs enthalpies of reaction compounds involved in MnO_2_ formation [32,33,34]. Formulas and oxidation states of all involved compounds are listed in Table A1.

Compound	Mn^2+^	Mn(OH)_2_	MnO_4_^−^	MnO_4_^2−^	MnO_4_^3−^	MnOOH	MnO_2_	Mn_3_O_4_	H_2_O	OH^−^
∆_r_G/kJ mol^−1^	−228	−615	−447	−501	−722	−548	−465	−1283	−237	−157

**Table 2:** Summary of AIB & ZIB (ChOAc & ZnSO_4_) redox potential region from half-cell measurements in Figure 8; * Extrapolated potential values based on measured redox potentials in ZIB.

Mn^x+^ Redox Pair		SHE	Al	Zn ChOAc	Zn ZnSO_4_
		V	V vs. Al/Al^3+^	V vs. Zn/Zn^2+^	V vs. Zn/Zn^2+^
Mn^3+^/Mn^2+^	Anodic	0.3–0.4	1.96–2.03	1.19	n.d.
	Cathodic	0.1–0.2	1.75–1.82	0.94	n.d.
Mn^4+^/Mn^3+^	Anodic	0.8–1.0	[2.47–2.67 *]	1.6–1.8	1.64
	Cathodic	0.6–0.7	[2.27–2.37 *]	1.4–1.5	1.28

* Bold marked potential values were calculated based on the measured peak potentials of Mn^4+^/Mn^3+^ in ZIB with CHOAc as electrolyte.

## Glossary

Interlayer distance = d-spacing:	distance from the manganese atom in the center of one oxide layer to the manganese atom of the next oxide layer
Gallery height = diffusion diameter:	Distance or height of the gap between two oxide layers

## Appendix A

**Table A1:** Overview of substances mentioned during this work.

Substance	Formula	Oxidation State
Birnessite	K_x_Mn(III)_x_Mn(IV)_1−x_O_4_ * y H_2_O	+III & + IV
Hausmannite	Mn_3_O_4_	+III & +II
Feitknechtite	MnOOH	+III
Permanganate	MnO_4_^−^	+VII
Manganate	MnO_4_^2−^	+VI
Hypomanganate	MnO_4_^3−^	+V
Manganese salt	Mn^2+^	+II

The star (*) in *birnessite* means that water molecules are located inside the crystal structure
